# The Complete Chloroplast Genome of *Catha edulis*: A Comparative Analysis of Genome Features with Related Species

**DOI:** 10.3390/ijms19020525

**Published:** 2018-02-09

**Authors:** Cuihua Gu, Luke R. Tembrock, Shaoyu Zheng, Zhiqiang Wu

**Affiliations:** 1 School of Landscape and Architecture, Zhejiang Agriculture and Forestry University, Hangzhou 311300, China; gu_cuihua@126.com (C.G.); aggies.collins@gmail.com (S.Z.); 2 Department of Biology, Colorado State University, Fort Collins, CO 80523, USA; Luke.R.Tembrock@aphis.usda.gov; 3 Department of Ecology, Evolution, and Organismal Biology, Ames, IA 50011, USA

**Keywords:** chloroplast (cp) genome, *Catha edulis*, next generation sequencing, phylogeny, repeat sequence

## Abstract

Qat (*Catha edulis*, Celastraceae) is a woody evergreen species with great economic and cultural importance. It is cultivated for its stimulant alkaloids cathine and cathinone in East Africa and southwest Arabia. However, genome information, especially DNA sequence resources, for *C. edulis* are limited, hindering studies regarding interspecific and intraspecific relationships. Herein, the complete chloroplast (cp) genome of *Catha edulis* is reported. This genome is 157,960 bp in length with 37% GC content and is structurally arranged into two 26,577 bp inverted repeats and two single-copy areas. The size of the small single-copy and the large single-copy regions were 18,491 bp and 86,315 bp, respectively. The *C. edulis* cp genome consists of 129 coding genes including 37 transfer RNA (tRNA) genes, 8 ribosomal RNA (rRNA) genes, and 84 protein coding genes. For those genes, 112 are single copy genes and 17 genes are duplicated in two inverted regions with seven tRNAs, four rRNAs, and six protein coding genes. The phylogenetic relationships resolved from the cp genome of qat and 32 other species confirms the monophyly of Celastraceae. The cp genomes of *C. edulis*, *Euonymus japonicus* and seven Celastraceae species lack the *rps16* intron, which indicates an intron loss took place among an ancestor of this family. The cp genome of *C. edulis* provides a highly valuable genetic resource for further phylogenomic research, barcoding and cp transformation in Celastraceae.

## 1. Introduction

Qat (Celastraceae: *Catha edulis* (Vahl) Forssk. ex Endl.) is a woody evergreen species of major cultural and economic importance in southwest Arabia and East Africa, which is cultivated for its stimulant alkaloids cathine and cathinone. An estimated 20 million people consume qat on a daily basis in eastern Africa [1], and its use and cultivation has been expanding in recent years [2]. Qat is the only species in Celastraceae that is cultivated on a large scale. The cultivation and/or collection (in some instances illegally from wild sources in protected areas) of qat takes place primarily in Israel, Ethiopia, Kenya, Madagascar, Rwanda, Tanzania, Somalia, Uganda, and Yemen [2,3,4].

The cultivation and sale of qat has become an important driver in the local and regional economies of East Africa and Yemen. In Yemen, 6% of the gross domestic product is generated from qat cultivation and sales [5]. Ethiopia has become the number one producer of qat in the world with exports in 1946 equaling only 26 tons valued at $5645, while 15,684 tons were exported in 2000 valued at $72 million [6]. A similar expansion in qat cultivation and sales has occurred in Kenya with the current trade from Kenya to Somalia estimated at $100 million per year. Trade of qat has become international in scale with, for example, 2.26 million kilograms of qat imported into England from Ethiopian and Kenya in 2013 [7]. The biosynthesis of cathinone and similar stimulant alkaloids is rare among green plants, known only in *Catha edulis* and several Asian species of *Ephedra* [8]. In addition, Celastraceae species produce numerous unique phytochemicals of potential pharmaceutical value [9]. Chloroplast transformations of qat and related species may prove useful for the production of cathinone related alkaloids and/or novel drugs.

The phylogenetic placement of qat within the Celastraceae has been inferred from 18S, 26S, *atpB*, ITS (as Nuclear ribosomal internal transcribed spacer), *matK*, *phyB*, and *rbcL* [10]. Phylogeographic work using SSR (as simple sequence repeats) loci has been done for wild and cultivated qat in the historic areas of production—Ethiopia, Kenya, and Yemen [7,11]. Beyond these studies, no genetic resources of which we are aware have been developed for qat. In addition, no chloroplast (cp) genome has been fully sequenced and published in the genus *Catha*. Therefore, our completed cp genome will be an important genetic resource for further evolutionary studies both within the Celastrales generally and economically important qat specifically.

The cp genome in plants is noted as being highly conserved in gene content [12]. Despite the consistency between cp genomes in plants, the differences in the size of cp genomes appear to be driven by intron and gene loss, and structural changes such as loss or gain of repeat units in different types of repetitive DNA [13]. In particular, genes that straddle inversion junctions such as *ycf1* appear to be undergoing rapid evolution [14].

Contrary to the structure of most nuclear plant genomes, the cp genome is typically comprised of a highly conserved quadripartite structure which is 115 to 165 kb in length, uniparentally inherited [12,15], and with similar gene content and order shared among most land plants [16]. From the advancements made by next-generation sequencing (NGS), complete, high quality cp genomes are becoming increasingly common [17]. At present, more than 2000 completed cp genomes of angiosperm species can be downloaded in the public database of the National Center for Biotechnology Information (NCBI; [18], Available online: https://www.ncbi.nlm.nih.gov/genomes/GenomesGroup.cgi?taxid=2759&opt=plastid). Large databases of complete cp genomes provide an indispensable resource for researchers identifying species [19], designing molecular markers for plant population studies, and for research concerning cp genome transformation [20,21,22]. The essentially non-recombinant structures of cp genomes make them particularly useful for the above applications. For example, cp genomes maintain a positive homologous recombination system [23,24,25,26]. Thus, in the transformation process, genes can be precisely transferred to specific genomic regions. A variety of homologous cp sites have proven useful at multiple levels of classification, including inter-specific and intra-specific [27]. In more recent years, systematic studies have employed entire cp genomes to attain high resolution phylogenies [28].

In this paper, we report the completely sequenced cp genome in the Celastrales and discuss the technical aspects of sequencing and assembly. In addition, we conduct phylogenetic analysis using other fully sequenced cp genomes from species in the closely related orders Malpighiales and Rosales. These analyses were conducted to find the top twenty loci for phylogenetic analysis and find which structural changes have taken place across cp genomes between the orders Rosales, Malpighiales, and Celastrales. The completed cp genome is a valuable resource for studying evolution and population genetics of both wild and cultivated populations of qat as well as genetic transformations related to the production of pharmaceuticals in qat or related Celastraceae species.

## 2. Results and Discussion

### 2.1. Chloroplast Assembly and Genome Features

The *C. edulis* cp genome was completely assembled into a single molecule of 157,960 bp, by combining Illumina and Sanger sequencing results. By mapping the completed genome using the paired reads, we confirm the size of our assembly for the completed cp genome with 497,848 (representing 5% of all reads) mapped pair-end reads evenly spanning the entire genome with mean read depth of 785× coverage (Figure S1). Given these quality controls and processing steps, the cp genome for qat is high quality.

Although the genome structure is highly conserved in the cp genome, several features such as the presence or lack of introns, the size of the intergenic region, gene duplication, and the length, type and number of repeat regions can vary [29]. The complete *C. edulis* cp genome has the conserved quadripartite structure and size that resembles most land plant cp genomes which are normally 115–165 kb in size including two inverted repeats (IRs) and two single-copy regions as large single copy and small single copy (LSC and SSC).

**Figure 1:**
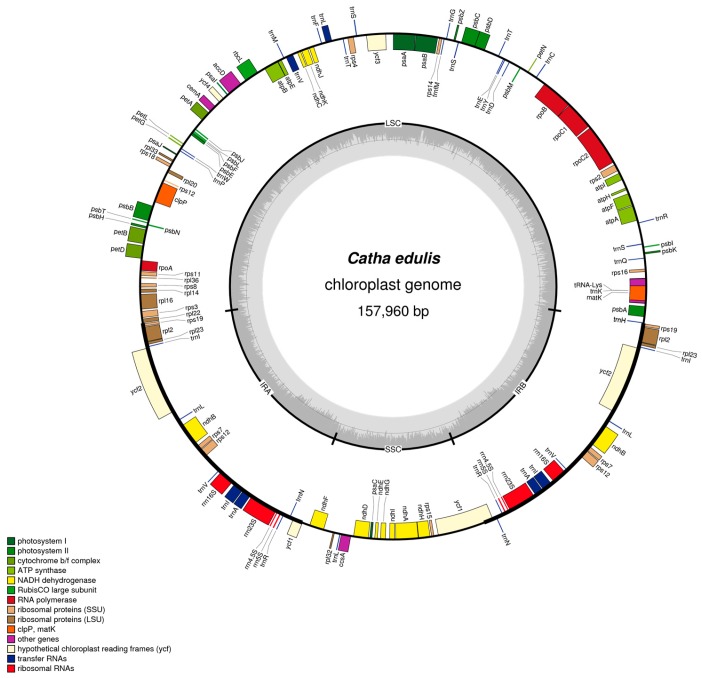
Circular map of the *C. edulis* cp genome. Genes shown inside and outside of the outer circle are transcribed clockwise and counterclockwise, respectively. The innermost shaded area inside the inner circle corresponds to GC content in the cp genome. Genes in different functional groups are color coded. IR, inverted repeat; LSC, large single copy region; SSC, small single copy region. The map is drawn using OGDRAW (V 1.2, Max Planck Institute of Molecular Plant Physiology, Am Mühlenberg, Germany).

The cp genome of *C. edulis* consists of two single-copy regions isolated by two identical IRs of 26,577 bp each, one SSC region of 18,491 bp and one LSC region of 86,315 bp. The proportion of LSC, SSC, and IRs size in the entire cp genome is 54.6%, 11.7% and 33.6%, respectively (Figure 1 and Table 1). The GC contents of the LSC, IR, SSC, and the whole cp genome are 35.1%, 42.7%, 31.8%, and 37.3%, respectively, which are consistent with the published Rosid cp genomes [30].

The *C. edulis* cp genome is composed of tRNAs, protein coding genes and rRNAs, intergenic and intronic regions (Table 2). Non-coding DNA accounts for 67,633 bp (42.8%) of the whole *C. edulis* cp genome, protein-coding genes account for 78,471 bp (49.7%), tRNA accounts for 2806 bp (1.8%), and rRNA accounts for 9050 bp (5.7%). By comparison with seven other species, gene order, gene content, the coding genes, and non-coding region proportions are similar among these cp genomes (Table 2).

### 2.2. Gene Content and Structure

The cp genome of *C. edulis* consisted of 129 coding regions made up of 37 tRNAs, 84 protein-coding genes, and eight rRNAs, of which 112 genes are unique and 17 genes were repeated in two inverted regions consisting of seven tRNAs, six protein coding genes, and four rRNAs (Figure 1 and Table 3). Among these 112 unique genes, three genes crossed different cp boundaries: *trnH^GUG^* crossed the IR_B_ and LSC regions, *ycf1* crossed the IR_B_ and SSC regions, *rps12* crossed two IR regions and the LSC region (two 3′ end exons repeated in IRs and 5′ end exon situated in LSC) (Figure 1). Of the remaining 109 genes, 80 are situated in LSC including 59 protein coding genes and 21 tRNAs, 17 in two inverted repeats (six coding genes, seven tRNAs, and four rRNAs), and 12 in the SSC including 11 coding genes and one tRNA.

Most of the protein-coding genes contain only one exon, while 17 genes contain one intron, of which four occur in both IRs, 12 genes are distributed in LSC, and one in the SSC (Table 4), among them three genes (*rps12*, *clpP* and *ycf3*) contain two introns, while 14 genes (*trnA^GUC^*, *trnI^GAU^*, *trnG^UCC^*, *trnL^UAA^*, *trnK^UUU^*, and *trnV^UAC^*, *rpoC1*, *atpF*, *rpl16*, *rpl2*, *petB*, *petD*, *ndhA*, and *ndhB*) contain one intron. The longest intron of *trnK^UUU^* is 2495 bp including the 1533 bp encoding the *matK* gene [13]. The *rps12* gene was predicted to be trans-spliced with a repeated 3′ end duplicated in two IRs and a single 5′ end exon in LSC [31].

### 2.3. Comparison of the cp Genomes

The cp genome of *C. edulis* (Celastraceae) was compared to species from 14 genera, including *Populus*, *Salix*, *Viola*, *Hevea*, *Manihot*, *Ricinus*, *Euonymus* and seven out-group species using dot-plot analysis. Besides a unique rearrangement of one 30-kb inversion in the *H. brasiliensis* cp genome [32], no other large structural differences (inversions) were detected among all compared species in the dot-plot analysis. This is consistent with the extremely conserved cp genomes in land plants [16]. The limited structural differences across the 14 species cp genomes demonstrate that gene order, gene content, and entire genome structure are conserved (Figure S3).

Based on the limited structural variation of cp genomes, we focused on seven closely related species of *C. edulis* to examine finer scale structural differences in genome length. Among these seven cp genomes, the length of genomes ranged from 155,590 bp (*S. purpurea*) to 163,161 bp (*R. communis*). The length of the LSC region varied from 84,452 bp (*S. purpurea*) to 89,651 bp (*R. communis*), and from 16,220 bp (*S. purpurea*) to 18,816 bp (*R. communis*) in SSC, and from 26,404 bp (*V. seoulensis*) to 27,646 bp (*P. euphratica*) in the IR regions (Table 2).

The entire GC content of the complete *C. edulis* cp genome is 37.3%, with 33.6% GC content in IRs, 35.1% in LSC, and 31.8% in SSC. These GC contents are consistent with other published cp genomes [33]. The whole GC content in the two Celastrales and six cp genomes of Malpighiales species ranged from 35.7% to 37.3% of the total genome, with *R. communis* having the lowest and *C. edulis* and *E. japonicus* having the highest GC content (Table 1).

**Figure 2:**
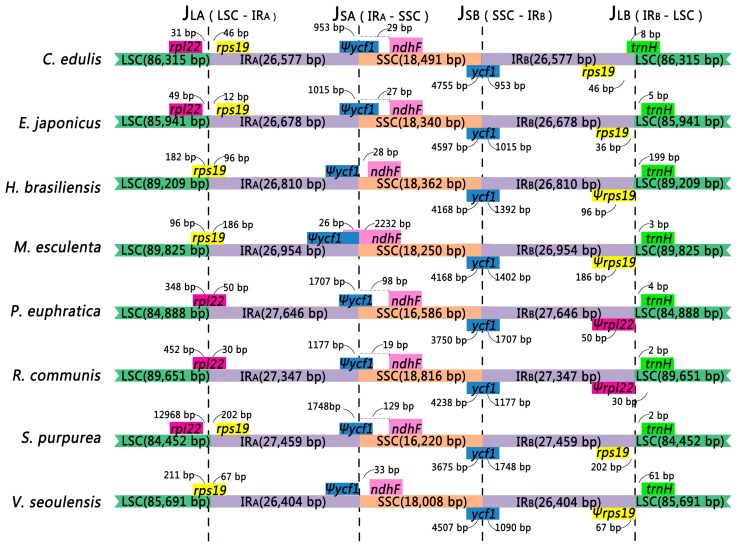
Comparison of junctions between the LSC, SSC, and IRs among eight species. Number above indicates the distance in bp between the ends of genes and the borders sites (distances are not to scale in this figure). The ψ symbol represents pseudogenes.

These eight species have similar genetic composition at the IR-SSC and IR-LSC boundaries except *rps19*, which is not present from the border of LSC and IR_A_ in *P. euphratica* and *R. communis* in which *rpl22* crosses the border of IR_A_ and LSC (Figure 2).

### 2.4. Contraction and Expansion in the Four Junction Regions

Although genomic structure including gene composition and genome size are highly conserved, expansion and contraction of IRs are common differences between plant cp genomes. Kim [34] proposed that the IRs size differ within plant cp genomes mainly results from the contraction or expansion at the junctions. Comparison of the inverted repeat-single copy (IR-SC) boundary regions of the two Celastrales and six Malpighiales species genomes showed very small differences in boundaries (Figure 2). We inspected the four boundaries (J_LA_, J_LB_, J_SA_, and J_SB_) across the two Celastrales and six Malpighiales species to detect the detailed boundary variation between the two SC regions and IRs using the methods described in [18].

The size of the IRs varied from 26,404 to 27,646 bp. The IR_A_-LSC junction (J_LA_) was situated in the *rps19* gene in *H. brasiliensis*, *M. esculenta*, and *V. seoulensis* which crossed inside the IR_A_ region 96 bp, 186 bp, and 67 bp, respectively, and as a result duplicated pseudogene *rps19* (ψ*rps19*) was nested within IR_B_ for these three species. However, in *C. edulis*, *E. japonicus* and *S. purpurea*, J_LA_ is situated in the intergenic regions between *rpl22* and *rps19* in which the distances from *rps19* to the J_LA_ were 46 bp, 12 bp and 202 bp. In two other species, *P. euphratica* and *R. communis*, J_LA_ is situated in the coding region of *rpl22* which spread into IR_A_ 50 bp and 30 bp, respectively, and resulted in the generation of pseudogene *rpl22* (ψ*rpl22*) in IR_B_.

The IR_A_-SSC junction (J_SA_) was situated in or adjoined pseudogene *ycf1* (ψ*ycf1*) for all eight species; J_SA_ of three species (*H. brasiliensis*, *M. esculenta*, and *V. seoulensis*) were all situated just adjacent to the end of ψ*ycf1*. Overlap between *ndhF* and ψ*ycf1* was found in *M. esculenta*, in which *ndhF* expanded into the IR_A_ region for 26 bp. For the other five species, J_SA_ was located near ψ*ycf1*. In the other six species (*C. edulis*, *E. japonicus*, *H. brasiliensis*, *P. euphratica*, *R. communis*, *S. purpurea* and *V. seoulensis*), the distances between *ndhF* and J_SA_ were 29 bp, 27 bp, 28 bp, 98 bp, 19 bp, 129 bp and 33 bp, respectively.

The IR_B_-SSC junction (J_SB_) is situated in the *ycf1* coding region which spans into the IR_B_ region in all eight species. However, the length of *ycf1* in the IR region varied among the eight species from 953 bp to 1748 bp highlighting the dynamic variation of the junction regions.

The IR_B_-LSC junctions (J_LB_) were located between *rps19* and *trnH* in *E. japonicus* and *S. purpurea*; situated at the end of ψ*rps19* in *H. brasiliensis*, *M. esculenta*; and *V. seoulensis*; and at the end of ψ*rpl22* in *P. euphratica* and *R. communis.* In the J_LB_ junction, the *trnH* gene is 8 bp into IR_B_ region in *C. edulis.* In the other seven species, 2–199 bp distance is found between the *trnH* gene and the IR_B_-SSC junction.

The variation in the IR-SC boundary area is due to the contraction or expansion of the IR observed in the IR-SSC boundaries. These expansions/contractions are likely to be mediated by molecular recombination within the two short, straight repeating sequences that occur frequently in the genes within the boundary [34].

### 2.5. Verification of the rps16 Intron Loss from Catha and Seven Other Celastraceae Species

The gene composition in the *C. edulis* cp genome is similar to the other angiosperm species analyzed in this study. However, we found that the *rps16* gene had no intron in the *C. edulis* cp genome. The structure and the intron size for *rps16* are conserved in the model species *Arabidopsis thaliana* and in our sampled species (NC_000932). However, it has been reported that *rps16* gene or the intron of *rps16* has been lost multiple times in numerous lineages [35,36].

**Figure 3:**
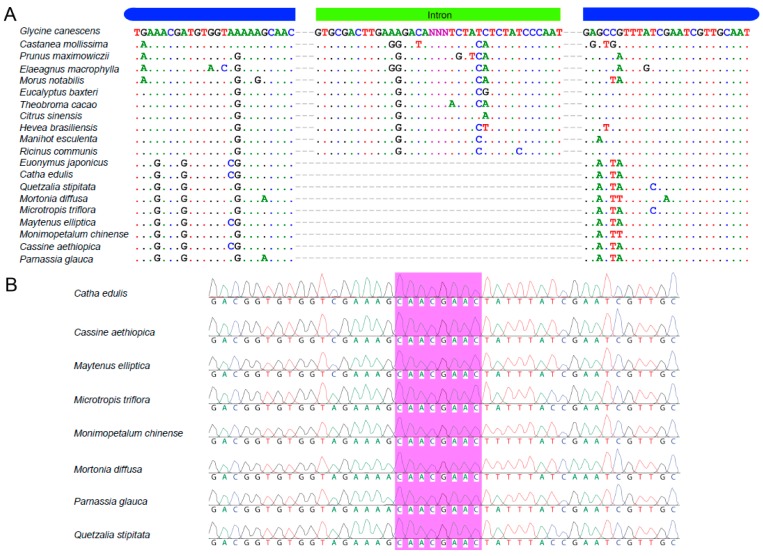
The sequence variation for *rps16* gene with and without intron: (**A**) The structural components of *rps16* gene in 20 species. All Species outside of Celastraceae family contained the *rps16* intron. (**B**) The purple area in all eight species from different genera of the Celastraceae family showed the connection of two exons indicating the lost intron.

To test whether the loss of the *rps16* intron is common throughout the Celastraceae family or just in certain species, two primers were designed in the flanking exons to amplify and then sequence the intron region (or lack thereof) for eight species in the Celastraceae family. Based on the PCR amplification (Figure S2), the length of this *rps16* amplicon is about 550 bp in all eight sampled Celastraceae species indicating that the intron has been lost throughout the Celastraceae family. We also conducted Sanger sequencing to verify the alignment of the *rps16* gene (Figure 3). From this alignment, all species sampled from the Celastraceae family do not contain the *rps16* intron (Figure 3A). The Sanger sequencing data provide additional evidence that all eight-species do not have this intron (Figure 3B).

Intron loss in cp genomes have been reported multiple times in different species, such as species in Desmodieae (Fabaceae) [37] and reported in both dicots and monocots. Loss of the *rps16* intron could probably be best explained by a homologous recombination and the reverse-transcriptase mediated mechanism [35]. However, intron loss from DNA fragment deletions or gene transfer between introns could be due to yet unexplained processes [37]. By increasing the sampling density within Celastraceae and its closest relatives, the timing of the *rps16* intron loss was inferred to occur between the Celastrales and Oxalidales + Malpighiales approximately 80 million years ago [38].

### 2.6. Identification of Long Repetitive Sequences

Long repetitive sequences play key functions in cp genome evolution, genome rearrangements and can be informative in phylogenetic studies [39]. Comparison of forward, complement, reverse, and palindromic repeats (≥30 bp) (with a sequence identity of ≥90% per repeat unit) were conducted across *C. edulis* and seven related species using REPuter (Available online: https://bibiserv.cebitec.uni-bielefeld.de/reputer/; (University of Bielefeld, Bielefeld, Germany)). *Catha edulis* had the fewest (8) repeats while its cp genome was not the shortest among those examined (157,960 bp) which is inconsistent with the general trend of shorter genomes possessing fewer repetitive regions [40].

**Figure 4:**
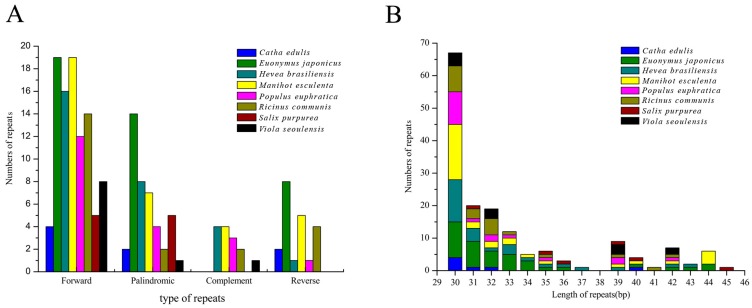
Analysis of repeat sequences in eight chloroplast genomes: (**A**) frequency of repeat types; and (**B**) frequency of the repeats by length ≥30 bp.

A total of 175 unique repeats consisting of forward, reverse, complementary and palindromic were found from the eight-species examined (Figure 4A). The species *E. japonicus* included the most repeats consisting of: 14 palindromic repeats, 19 forward repeats, and eight reverse repeats, for a total of 41 repeats (Figure 4A and Table S3). In *H. brasiliensis*, *M. esculenta*, *P. euphratica*, *R. communis*, *S. purpurea* and *V. seoulensis* cp genomes, 29, 35, 20, 22, 10, and 10 total repeat pairs were found respectively (Figure 4A). Among them, 19 forward repeats were most commonly found in *E. japonicus* and *M. esculenta* and in all species the most common repeat type was forward (Figure 4A). Forward repeats are often the result of transposon activity [41], which can increase under cellular stress [42]. However, the origins and multiplication of long repetitive repeats is not fully understood [43]. Previous studies suggested that the existence of genome rearrangement could be attributed to slipped-strand mispairing and inapposite recombination of repetitive sequences [43]. Moreover, forward repeats can lead to changes in genomic structure and thus be used as markers in phylogenetic studies. The length of repeats is variable in this study, with the shortest at 30 bp and the longest at 95 bp (Table S3). The majority of repeats (82%) varied from 30 bp to 40 bp in length (Figure 4B and Table S3). Given the variability of these repeats between lineages, they can be informative regions for developing genomic markers for population and phylogenetic studies [44].

### 2.7. Chloroplast Genome Simple Sequence Repeats (SSRs)

Simple sequence repeats (SSRs) are sequences with motifs from 1 to 6 bp in length repeated multiple times (see methods for cutoff criteria), are found distributed throughout the cp genome, and are often used as markers for breeding studies, population genetics, and genetic linkage mapping [43,45].

**Figure 5:**
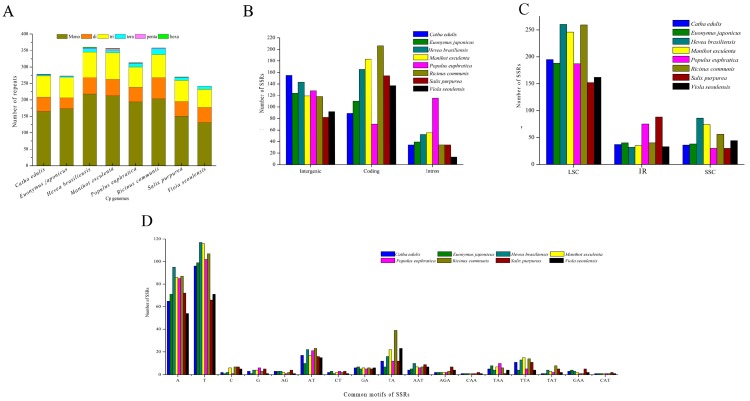
The distribution, type, and presence of simple sequence repeats (SSRs) in eight chloroplast genomes: (**A**) number of different SSR types detected in eight chloroplast genomes presence of SSRs at the LSC, SSC, and IR regions.; (**B**) frequency of SSRs in the protein-coding regions, intergenic spacers and intronic regions; (**C**) frequency of SSRs in the LSC, SSC, and IR regions; and (**D**) frequency of common motifs in the eight chloroplast genomes.

A total of 278 SSRs were found in the *C. edulis* cp genome (Figure 5A and Table S4). These SSRs include 165 mononucleotide SSRs (59%), 43 dinucleotide SSRs (15%), 65 trinucleotide SSRs (23%), 3 tetranucleotide (0.01%), and 1 pentanucleotide SSR (0.003%) (Figure 5A and Table S4). Among the 165 SSRs, 98% of SSRs (161) are the AT type with copy number from 8 to 18 (Table S4). In these SSRs of the *C. edulis* cp genome, 89 SSRs were detected in protein-coding genes, 34 SSRs in introns, and 155 in intergenic regions (Figure 5B). In relation to the quadripartite, 195 SSRs were situated in the LSC, whereas 36 and 37 were identified in the SSC and IR, respectively (Figure 5C).

Among the eight species, *V. seoulensis* had the fewest SSRs (242) and *H. brasiliensis* had the most SSRs (360). *Salix purpurea* has the shortest cp genome (155,590 bp) with 270 SSRs and *R. communis* has the longest cp genome (163,161 bp) and 358 SSRs of those analyzed in this study suggesting that number of SSRs may affect genome length, but a strong correlation was not found in all species (Figure 5A). This result indicates that cp genome sizes were not obviously connected with the number of SSRs in these species. Additionally, an abundance of tetranucleotide SSRs were not found in the species studied and no pentanucleotide SSRs were found in *V. seoulensis* or hexanucleotide in *E. japonicus*, *R. communis* and *V. seoulensis* (Figure 5A). Among the eight species, most SSRs of *C. edulis* and *E. japonicus* were located in intergenic regions, most SSRs of *H. brasiliensis*, *M. esculenta*, *P. euphratica*, *R. communis*, and *V. seoulensis* in coding regions, and most SSRs of *S. purpurea* are in intronic regions (Figure 5B). Some SSRs were distributed in protein-coding regions such as *ycf1* and *rpoC2* (Table S4), which could also be employed as DNA markers for population level and genomic studies. Most SSRs in all eight-species were in the LSC region (Figure 5C). Common motifs in the eight-species studied generally consisted of polythymine (poly-T) or polyadenine (poly-A) (Figure 5D). The Euphorbiaceae species in this study all have more SSRs than the other species in this study as well as similar patterns of distribution in the genome. More work is needed to understand these patterns of SSR distribution in cp genomes. Lastly, the SSRs from this study should be valuable for phylogeographic studies and comparing phylogenetic relationships among Celastraceae species.

### 2.8. Highly Informative Coding Genes and Markers for Phylogenomic Analysis

Detecting highly informative and variable coding genes is important for DNA barcoding, marker development and phylogenomic analyses [46]. Coding genes such as *matK*, *rbcL* have been widely employed for barcoding applications [47,48] and phylogenetic reconstructions [49,50,51]. Based on compared complete cp genomes, additional informative markers were identified within the Celastraceae.

We aligned entire coding genes more than 200 bp in length to discover genes with the highest sequence identity index and the highest proportion of parsimony-informative sites, for the seven species in this study (Table 5, Table S5). In the coding regions, *matK* and *ycf1* have the largest proportion of parsimony information characters (16.83% and 16.80%, respectively). The *matK* gene is used as core DNA barcoding sequence under the suggestion of CBOL working group (CBOL is The Consortium for the Barcode of Life, an international initiative devoted to developing DNA barcoding as a global standard for the identification of biological species) and also in concert with other variable genes such as *ITS* + *psbA-trnH* + *matK* which was shown to have the highest species identification rate [52]. Given the high number of parsimony informative in *ycf1*, it may also serve as another core DNA barcode in future plant studies [14]. The coding regions identified in this analysis (Table 5) should be particularly informative for species identification and phylogenetic analyses due to the high percentage of variable sites.

### 2.9. Phylogenetic Analysis

**Figure 6:**
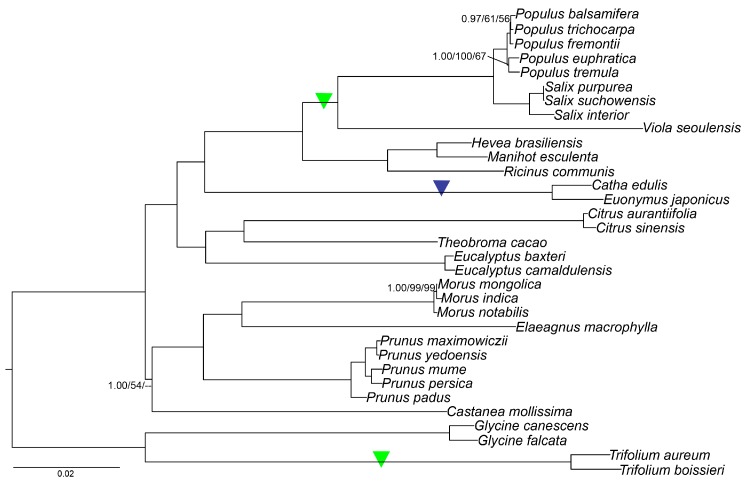
Phylogenetic tree based on 73 shared protein-coding genes was constructed for 33 species using three different methods, including Parsimony analysis, maximum likelihood (ML) and Bayesian inference (BI). All branches had bootstrap values or posterior probability of 100/1.00 except those labeled. The *rps16* gene losses are indicated with green triangles and the *rps16* intron loss is indicated with a purple triangle.

Based on cp genomes, phylogenetic analyses have helped to resolve the relationships of many angiosperm lineages [53,54]. Previous phylogenetic work in Celastraceae was inferred based on nuclear (26S rDNA and ITS) together with morphological traits and chloroplast genes (*matK*, *trnL-F*) [10]. Our phylogenetic analyses included *C. edulis* and 28 species which were sampled based on relationships from NCBI database (Available online: http://www.ncbi.nlm.nih.gov/genomes/GenomesGroup.cgi?taxid=2759&opt=plastid) and the angiosperm tree of life (Available online: http://www.mobot.org/mobot/research/apweb/) with *Glycine canescens*, *Glycine falcate*, *Trifolium aureum*, and *Trifolium boissieri* from Fabaceae as outgroup taxa. The phylogenetic tree indicated that *Catha* and *Euonymus* where most closely related based on 73 common protein-coding genes (Figure 6). Most branches of the phylogenetic tree had high bootstrap support with all three methods. This suggests that the full cp genome information could be very useful in resolving phylogenetic conflicts but phylogenetic analyses with many closely related species are needed to test the resolving power of chloroplast coding genes [55].

With a clearly resolved and strongly supported phylogeny, evolutionary patterns can be more clearly interpreted, such as gene or intron sequence loss/gain. Specifically, the intron loss of the *rps16* gene and loss of the whole *rps16* gene (Figure 6), were found in Celastraceae (*rps16* intron loss) and independently (*rps16* gene loss) in the genus *Trifolium* (Fabaceae), and the clade Salicaceae + Violaceae (Table S6). Gene and intron loss have been noted numerous times in land plant cp genomes [37]. From the phylogenetic tree, we were able to infer that the intron of *rps16* was lost in an ancestor to the Celastraceae independently from the two *rps16* gene loss events (Figure 6). Why only the *rps16* intron was lost in the Celastraceae and the entire gene in other closely related lineages is not known. Further study is needed to understand the underlying mechanisms of gene vs. intron loss in these related groups.

## 3. Materials and Methods

### 3.1. DNA Extraction and Sequencing

DNA for this project was obtained from aliquots of the extracts used in Tembrock et al., 2017. Total genomic DNA was used to build sequence libraries (Illumina Inc., San Diego, CA, USA), and was extracted from leaves using a *Catha* specific DNA extraction protocol described in Tembrock et al., 2017. At the Beijing Genomics Institute (BGI), an Illumina HiSeq 2000 sequencer was used to sequence paired-end (PE) sequencing libraries with an average 300 bp insert length. From this, over 10 million clean reads were passed through quality control with a 100 bp each read length. All other used species in this paper were listed in Table S1.

### 3.2. Chloroplast Genome Assembly and Sequence Analysis

The original Illumina reads were pre-processed, including the trimming and filtering of low-quality sequences with Trimmomatic v0.3 [56] in which the parameters used were as follows: minlen: 50; trailing: 3; leading: 3; and sliding window: 4:15. De novo assembly from *C. edulis* employed the default parameters (Available online: http://www.clcbio.com) in the CLC genomic workbench v7 (CLCbio, Hilden, Germany). Then, three independent de novo assemblies, which included single-end forward reads, single end reverse reads, and PE reads, were performed [18]. After that, a single assembly formed by the combination of these three separate assemblies was conducted. From the complete CLC assembly results, assembled contigs longer than 0.5 kb with over 100× coverage were compared to complete cp genomes of several species, including *Euonymus japonicus* (Celastraceae, KP189362), *Populus euphratica* (Salicaceae; NC_024747), and *Salix purpurea* (Salicaceae; NC_026722). Matching the contigs from the cp genomes was done using Local BlastN searches [57]. Using the conserved cp genome regions, the related cp genomes were matched with the mapped contigs [58] and then a single contig was connected to these contigs to create the quadripartite genome employing Contig Express 2003 (Invitrogen, Carlsbad, CA, USA). By designing primers in regions flanking gaps, PCR amplification was carried out and the gap sequences were completed by adding sequence data obtained from Sanger sequencing (Figure S2).

Additionally, primers were designed to verify de novo sequence assemblies, such as the junction regions of the cp genome (Table S2). The 40-μL PCR volume was setup as follows: 10× Taq buffer 4 μL, ddH_2_O 33.3 μL, 10 mM dNTP 0.8 μL, 20 pmol/μL each primer 0.5 μL, 5 U/μL Taq polymerase 0.4 μL and DNA template 0.5 μL. Taq buffer, dNTP, primers were from Sangong Biotech (Shanghai, China). Cycling conditions were 94 °C for 5 min, 32 cycles 94 °C for 45 s, 54 °C for 45 s, 72 °C for 2 min and, a 10 min 72 °C final extension step. By combining the results of Sanger sequencing, the whole cp genome was used to map reference species to confirm the assembly with the uniformity of the iterative sequences.

Annotation of the transfer RNAs (tRNAs), protein-coding genes, and ribosomal RNAs (rRNAs) was first performed using DOGMA v1.2 (University of Texas at Austin, Austin, TX, USA) [59]. Then, the protein-coding gene positions in the draft annotation were verified and if necessary manually adjusted following alignment to the related species, *Euonymus japonicas* [58] to accurately determine the genes starting point, stop codons and exon borders. Finally, BLASTN searches and tRNAscan-SE v1.21 (University of California Santa Cruz, CA, USA) [60] were employed to verify both tRNA and rRNA genes.

A graphical cp genome map for *C. edulis* was completed using OGDraw (OrganellarGenomeDRAW) (V 1.2, Max Planck Institute of Molecular Plant Physiology, Am Mühlenberg, Germany) [61]. The annotated *C. edulis* cp genome reported and analyzed herein has been deposited in GenBank (KT861471).

### 3.3. Chloroplast Genomes Comparison

#### 3.3.1. IR Expansion and Contraction

The changes in the size of the angiosperm cp genomes are mainly due to the contraction and expansion from the inverted repeat region, and the two single copy boundary areas. Four borders (J_LA_, J_LB_, J_SA_, and J_SB_) are present in the *C. edulis* cp genome and are situated in the middle of two IRs and two single copy regions [62]. The IR borders and neighboring genes of the two Celastrales species (*Catha edulis* and *Euonymus japonicus*) and six Malpighiales species cp genomes (*Hevea brasiliensis*, *Manihot esculea*, *Populus euphratica*, *Ricinus communis*, *Salix purpurea*, and *Viola seoulensis*) were compared in this study.

#### 3.3.2. Repeat Analysis

Two methods were used to search repeats in *C. edulis* [63]. We identified simple sequence repeats (SSRs) using SSR Hunter v1.3 (Nanjing Agricutural University, Nanjing, China) [64] with cut-offs of eight copy number for mono-SSRs, four copy number for di-, three copy number for tri-, tetra-, penta- and hexanucleotide SSRs. To discover larger repeat regions, REPuter [65] was employed to find four possible repeats types: containing complement, forward, palindrome, and reverse repeats. Nested and low complexity repeats were not included in this study [66].

#### 3.3.3. Dot-Plot Analysis

To identify the structural variations across all 14 genera, *Populus* (Salicaceae; Malpighiales), *Salix* (Salicaceae; Malpighiales), *Viola* (Violaceae; Malpighiales), *Hevea* (Euphorbiaceae; Malpighiales), *Manihot* (Euphorbiaceae; Malpighiales), *Ricinus* (Euphorbiaceae; Malpighiales), and *Euonymus* (Celastraceae; Celastrales), as well as outgroup genera *Prunus*, *Morus*, *Theobroma*, *Eucalyptus*, *Elaeagnus*, *Castanea*, and *Citrus*, we conducted the dot-plot analysis (based on a custom perl script) [13] between *C. edulis* and all 14 genera to visualize structural differences in two dimensional plots.

#### 3.3.4. Verification of the *rps16* Intron Loss from Catha and Seven Other Celastraceae Genera

During annotation, the intron loss of *rps16* was found in the cp genome of *C. edulis*. To verify whether this intron loss happened throughout Celastraceae, two primers were designed (Forward-ACTTCGTTTGAGACGGTGTG, Reverse- AAAAACCCCGATTTCTTTGA) to amplify the entire *rps16* intron from *C. edulis* and seven other Celastraceae species (*Quetzalia stipitata*, *Mortonia diffusa*, *Microtropis triflora*, *Maytenus elliptica*, *Monimopetalum chinensis*, *Cassine aethiopica*, and *Parnassia glauca*). In *C. edulis*, the target *rps16* fragment without the intron is about 550 bp. Absence of the *rps16* intron was visualized on 0.8% agarose gels. The size of the fragment was determined by comparing it to a DNA size standard [67]. The *rps16* gene was sequenced using Sanger sequencing at the Beijing Genomics Institute (BGI).

#### 3.3.5. Phylogenetic Analyses

The 73 common protein-coding genes of 26 species cp genomes, among them eight Rosales and four Fabales outgroup species, were aligned under the default parameters of Clustal X, with reading frames included by manual correction (Supplement data matrix) [68]. The phylogenetic tree based on these 73 common genes was inferred using three different methods. Implementation of Parsimony analysis, Bayesian inference (BI), and maximum likelihood (ML) were made in PAUP* 4.0b10 [69], MrBayes 3.1.2, and PHYML v 2.4.5 [70,71] respectively using the parameters from Wu et al. [18].

## 4. Conclusions

In this study, using next generation sequencing technology, we successfully completed the whole chloroplast genome for the economically important species *C. edulis*. In comparing the *C*. *edulis* cp genome with numerous closely related species, we found that it has a typical angiosperm cp genome structure and gene content. However, some unique features are reported here, such as the loss of the intron region from the *rps16* gene, and repeat structure and abundance. We also resolved the phylogenetic position of *C. edulis* with its relatives including the monophyly of Celastraceae. The whole cp genome of *C. edulis* provides a valuable genetic resource for further phylogenomic research, barcoding, and cp transformation in Celastraceae.

## Figures and Tables

**Table 1:** Comparison of plastid genome size among eight species.

Region	Features	*C. edulis*	*E. japonicus*	*H. brasiliensis*	*M. esculenta*	*P. euphratica*	*R. communis*	*S. purpurea*	*V. seoulensis*
LSC	Length (bp)	86,315	85,941	89,209	89,295	84,888	89,651	84,452	85,691
	GC Content (%)	35.1	35.1	33.2	33.3	34.5	33.3	34.4	33.8
	Length Percentage (%)	54.6	54.5	55.3	55.3	54.1	54.9	54.3	54.8
SSC	Length (bp)	18,491	18,340	18,362	18,250	16,586	18,816	16,220	18,008
	GC Content (%)	31.8	31.8	29.5	29.6	30.6	29.5	31	29.6
	Length Percentage (%)	11.7	11.6	11.4	11.3	10.6	11.5	10.4	11.5
IR	Length (bp)	26,577	26,678	26,810	26,954	27,646	27,347	27,459	26,404
	GC Content (%)	42.7	42.7	42.2	42.3	41.9	41.9	41.9	42.6
	Length Percentage (%)	16.8	16.9	16.6	16.7	17.6	16.8	17.6	16.9
Total	Length (bp)	157,960	157,637	161,191	161,453	156,766	163,161	155,590	156,507
	GC Content (%)	37.3	37.3	35.7	35.9	36.7	35.7	36.7	36.3

LSC, large single copy region; SSC, small single copy region; IR, inverted repeat.

**Table 2:** Comparison of coding and non-coding region size among eight species.

Region	Species	*C. edulis*	*E. japonicus*	*H. brasiliensis*	*M. esculenta*	*P. euphratica*	*R. communis*	*S. purpurea*	*V. seoulensis*
Protein coding	length (bp)	78,471	77,331	78,852	79,089	78,728	78,119	77,898	78,310
	Length Percentage (%)	49.7	49.1	48.9	49.0	50.2	47.9	50.1	50.0
	GC Content (%)	38	38.2	37.1	37.2	37.6	37.5	37.6	37.2
tRNA	length (bp)	2806	2806	2798	2742	2796	2802	2792	2810
	Length Percentage (%)	1.8	1.8	1.7	1.7	1.8	1.7	1.8	1.8
	GC Content (%)	52.6	53.3	53.2	53.3	53	53.2	52.9	53
rRNA	length (bp)	9,050	9050	9050	9050	9050	9050	9,050	9050
	Length Percentage (%)	5.7	5.7	5.6	5.6	5.8	5.5	5.8	5.8
	GC Content (%)	55.2	55.4	55.4	55.5	55.5	55.5	55.4	55.4
Intron	length (bp)	18,474	19,287	18,538	18,479	18,210	18,278	17,321	18,348
	Length Percentage (%)	11.7	12.2	11.5	11.4	11.6	11.2	11.1	11.7
	GC Content (%)	37.1	36.6	36.6	36.9	36.9	37.1	37.3	36.7
Intergenic	length (bp)	49,159	49,163	51,953	52,093	47,982	54,912	48,529	47,989
	Length Percentage (%)	31.1	31.2	32.2	32.3	30.6	33.7	31.2	30.7
	GC Content (%)	31.9	31.7	29	29	31	28.7	30.7	30.1

**Table 3:** List of genes in the *C. edulis* plastid genome.

Gene Category	Groups of Genes	Name of Genes
Self-replication	Transfer RNA genes	*trnA^UGC^* ^a,b^ *trnC^GCA^ trnD^GUC^ trnE^UUC^ trnF^GAA^ trnfM^CAU^ trnG^UCC^ trnG^GCC^ trnH^GUG^ trnI^CAU^* ^b^ *trnI^GAU^* ^a,b^ *trnK^UUU^* ^a^ *trnL^CAA^* ^b^ *trnL^UAA^* ^a^ *trnL^UAG^ trnM^CAU^ trnN^GUU^* ^b^ *trnP^UGG^ trnQ^UUG^ trnR^ACG^* ^b^ *trnR^UCU^ trnS^GCU^ trnS^GGA^ trnS^UGA^ trnT^GGU^ trnT^UGU^ trnV^GAC^* ^b^ *trnV^UAC^* ^a^ *trnW^CCA^ trnY^GUA^*
	Small subunit of ribosome	*rps2 rps3 rps4 rps7b rps8 rps11 rps12* ^a,b^ *rps14 rps15 rps16 rps18 rps19*
	Ribosomal RNA genes	*rrn16* ^b^ *rrn23* ^b^ *rrn4.5* ^b^ *rrn5* ^b^
	Large subunit of ribosome	*rpl2* ^b^ *rpl14 rpl16* ^a^ *rpl20 rpl22 rpl23* ^b^ *rpl32 rpl33 rpl36*
	DNA dependent RNA polymerase	*rpoA rpoB rpoC1* ^a^ *rpoC2*
Photosynthesis	Subunits of photosystem I	*psaA psaB psaC psaI psaJ*
	Subunits of photosystem II	*psbA psbB psbC psbD psbE psbF psbH psbI psbJ psbK psbL psbM psbN psbT psbZ*
	Subunits of cytochrome	*petA petB* ^a^ *petD* ^a^ *petG petL petN*
	Subunits of ATP synthase	*atpA atpB atpE atpF* ^a^ *atpH atpI*
	ATP-dependent protease subunit p gene	*clpP* ^a^
	Large subunit of Rubisco	*rbcL*
	Subunits of NADH dehydrogenase	*ndhA* ^a^ *ndhB* ^a,b^ *ndhC ndhD ndhE ndhF ndhG ndhH ndhI ndhJ ndhK*
Other genes	Maturase	*matK*
	Envelop membrane protein	*cemA*
	Subunit of acetyl-CoA-carboxylase	*accD*
	c-type cytochrome synthesis gene	*ccsA*
Genes of unknown function	Conserved open reading frames	*ycf1 ycf2* ^b^ *ycf3* ^a^ *ycf4*

^a^ Genes containing introns; ^b^ Duplicated gene (Genes present in the IR regions).

**Table 4:** Genes with intron and their length of exons and introns in plastid genome of *C. edulis*.

Gene Name	Location	Exon I (bp)	Intron I (bp)	Exon II (bp)	Intron II (bp)	Exon III (bp)
*rpoC1*	LSC	1632	817	441		
*atpF*	LSC	396	699	159		
*petB*	LSC	6	773	642		
*petD*	LSC	8	784	475		
*ndhB*	IR	756	687	777		
*ndhA*	SSC	540	1178	573		
*rpl16*	LSC	399	1119	9		
*rpl2*	IR	471	648	393		
*rps12*	LSC	114		27	546	231
*ycf3*	LSC	153	727	228	731	126
*clpP*	LSC	231	676	291	849	69
*trnK-UUU*	LSC	29	2495	37		
*trnL-UAA*	LSC	37	540	50		
*trnV-UAC*	LSC	37	663	39		
*trnI-GAU*	IR	42	939	35		
*trnA-UGC*	IR	38	801	35		
*trnG-UCC*	LSC	23	761	48		

**Table 5:** Ten highest informative sites of coding genes in eight species.

No.	Region	Length (bp) ^1^	Aligned Length (bp) ^2^	Conserved Sites	Parsimony Informative ^3^	Parsimony Informative % ^4^	CI. ^5^	RI ^6^	SI ^7^
1	*matK*	1518	1575	1028	265	16.83	0.82	0.7	0.9
2	*ycf1*	5640	6327	3970	1063	16.80	0.82	0.6	0.8
3	*ccsA*	969	987	689	160	16.21	0.84	0.7	0.9
4	*accD*	1509	1401	242	227	16.20	0.83	0.7	0.8
5	*rps3*	648	663	467	107	16.14	0.82	0.7	0.9
6	*ndhF*	2232	2331	1606	368	15.79	0.81	0.6	0.8
7	*rps8*	405	411	294	64	15.57	0.8	0.7	0.9
8	*rpl22*	399	551	345	82	14.88	0.83	0.6	0.7
9	*petL*	96	96	70	14	14.58	0.9	0.8	0.9
10	*ndhD*	1503	1527	1116	207	13.56	0.82	0.7	0.9

^1^ Length: refers to sequence length in *Catha edulis*; ^2^ Aligned length: refers to the alignment of seven other species considered in the comparative analysis (see Materials and Methods); ^3^ Number of parsimony informative sites; ^4^ Percentage of parsimony informative sites; ^5^ CI: Consistency Index; ^6^ RI: Retention Index; ^7^ SI: Sequence Identity.

## Supplementary Materials

Supplementary materials can be found at http://www.mdpi.com/1422-0067/19/2/525/s1.
