# Supplementary figures and images for: The Complete Chloroplast Genome of Catha edulis: A Comparative Analysis of Genome Features with Related Species
Source: Int J Mol Sci. 2018 Feb 9;19(2):525. doi: 10.3390/ijms19020525 (PMC5855747; doi:10.3390/ijms19020525)

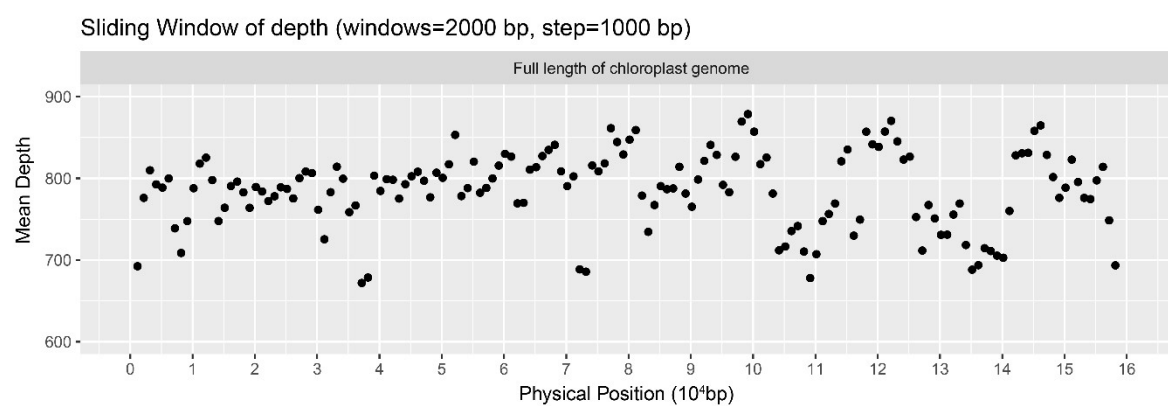

Figure S1

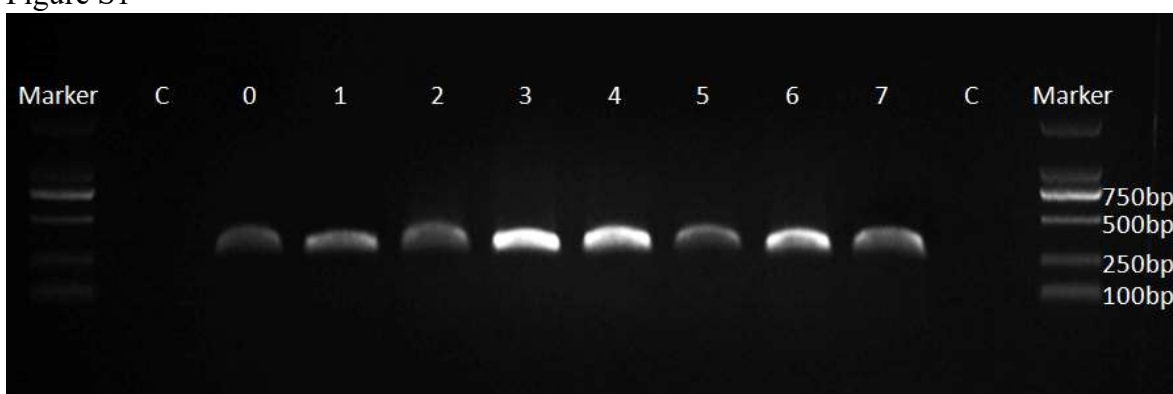

Figure S2

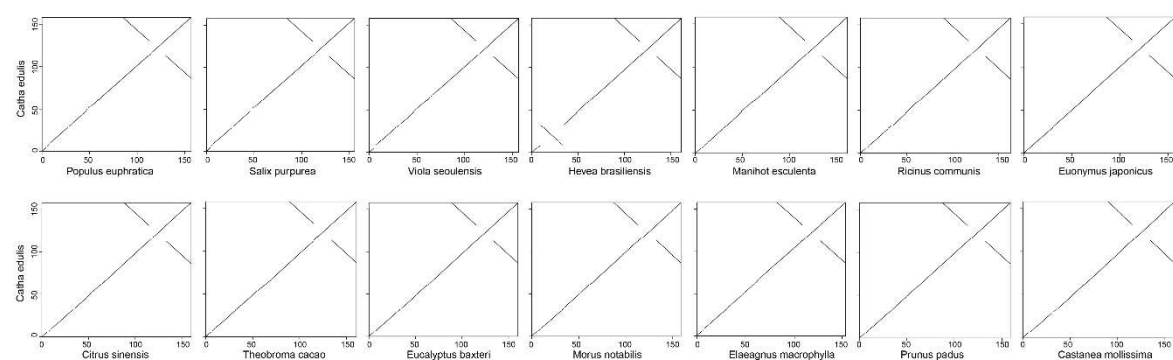

Figure S3

Supplement: Supplementary file 1 [file ijms-19-00525-s001.zip › ijms-258961-supplementary Files/figure S1-S3.pdf]

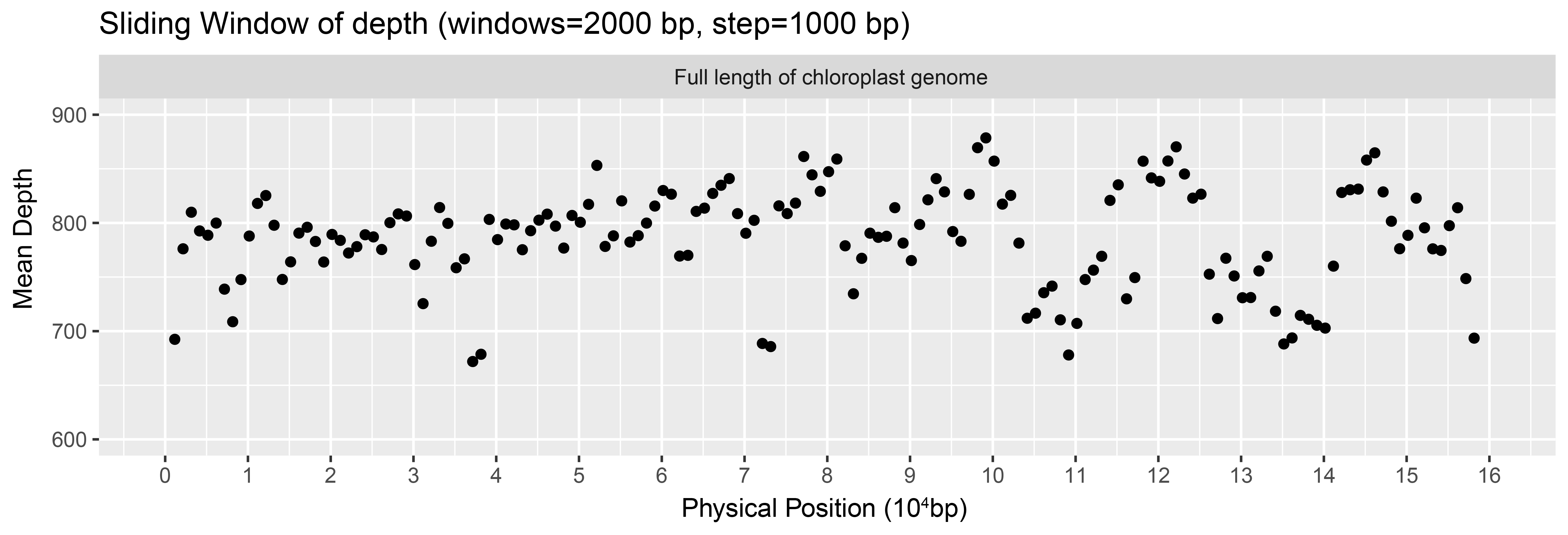

Supplement: Supplementary file 1 [file ijms-19-00525-s001.zip › ijms-258961-supplementary Files/Figure S1.tif]

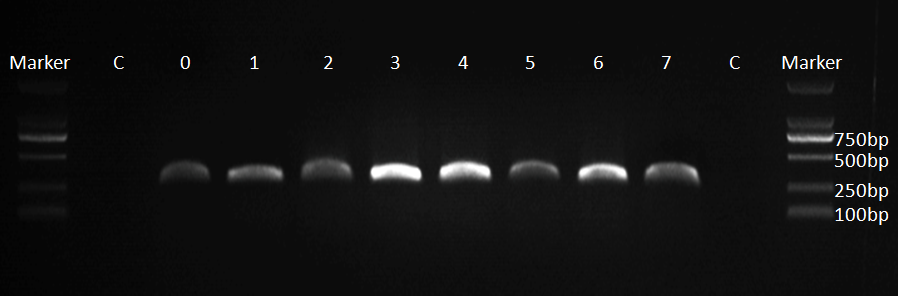

Supplement: Supplementary file 1 [file ijms-19-00525-s001.zip › ijms-258961-supplementary Files/Figure S2.tif]

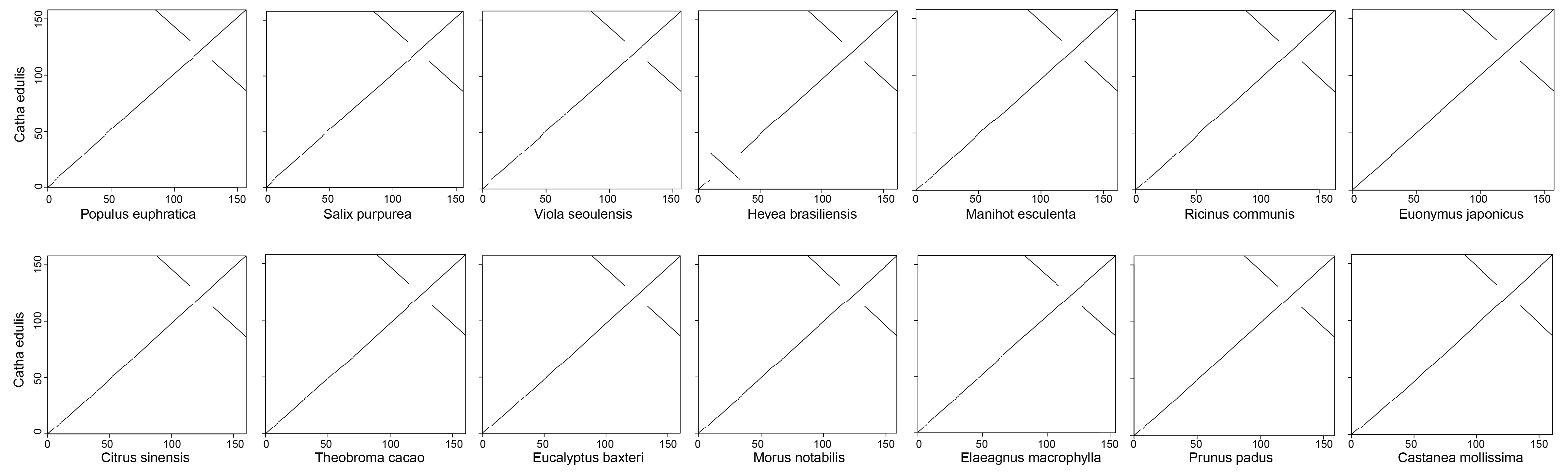

Supplement: Supplementary file 1 [file ijms-19-00525-s001.zip › ijms-258961-supplementary Files/Figure S3.tif]
